# 295. Humanized Mice and Convalescent Humans Yield an Antibody Panel against the Highly Immunogenic Decorin Binding Protein A (DbpA) of the Lyme Disease Spirochete, *Borrelia Burgdorferi*

**DOI:** 10.1093/ofid/ofad500.367

**Published:** 2023-11-27

**Authors:** Beatrice M Muriuki, Rachel M Golonka, Zepei Ma, Ravi Bharadwaj, Qi Li, David Vance, Graham G Willsey, Carol Lyn Piazza, Natalie J Rudolph, Nicholas Mantis, Yang Wang, Lisa Cavacini

**Affiliations:** MassBiologics of Umass Chan Medical School, Boston, Massachusetts; MassBiologics of UMass Chan Medical School, Boston, Massachusetts; MassBiologics, Boston, Massachusetts; UMass Chan Medical School, worcester, Massachusetts; MassBiologics, Boston, Massachusetts; Wadsworth Center, Albany, New York; Wadsworth Center, Albany, New York; Wadsworth Center, New York State Department of Health, Albany, New York; New York Structural Biology Center, New York, New York; Wadsworth Center, Albany, New York; MassBiologics, Boston, Massachusetts; MassBiologics, Boston, Massachusetts

## Abstract

**Background:**

Lyme disease is the most commonly diagnosed tick-borne infection, affecting more than 300000 people in the United States annually. The disease is principally caused by the transmission of the spirochete bacteria, by the black-legged (deer) ticks. With a renewed call for interest in a Lyme disease vaccine, antibodies relevance continues due to their efficacy, safety and versatile nature. Humanized mice and humans recovering from the disease have successfully been used to generate therapeutic antibodies against Ebola and SARS-CoV-2 diseases.

**Methods:**

Our study used humanized mice and convalescent patients to generate recombinant human antibodies that recognize decorin-binding protein A (DbpA), a lipoprotein that is proposed to function as spirochete adhesin. The mAbs expressed as human IgG-Fc fragments were characterized for reactivity with recombinant DbpA, native DbpA on the surface of live *Borrelia burgdorferi*, borreliacidal activity in the presence of human complement and, finally subject to epitope mapping using an array of methodologies.

**Results:**

The predominant lineage of humanized mice antibodies utilized VH5-51, VH3-20, VH3-23 and VH3-53 all paired with VK1-33 families. Other common families included VH4-31 paired with VK1-39, VH4-4 and VH4-8 paired with VK-9. The human derived antibody utilized VH3-30 paired with VK1-33. We observed an overlap in the repertoire of kappa chains between humanized mice and human-derived mAbs.

**Conclusion:**

Combining the humanized mice and human platforms enabled the identification of antibodies with a high binding and specificity to DbpA, which have potential prophylactic and therapeutic properties.

**Disclosures:**

**All Authors**: No reported disclosures

